# (4*S*)-(−)-4-Benzyl-2,2-dimethyl-3-*o*-toluoyl-1,3-oxazolidine

**DOI:** 10.1107/S1600536810040699

**Published:** 2010-10-20

**Authors:** Andrzej K. Gzella, Maria Chrzanowska, Agnieszka Dreas, Zofia Meissner

**Affiliations:** a Faculty of Pharmacy, Ludwik Rydygier Collegium Medicum in Bydgoszcz, Nicolaus Copernicus University in Torun, ul. M. Curie Skłodowskiej 9, 85-094 Bydgoszcz, Poland; b Faculty of Chemistry, A. Mickiewicz University, ul. Grunwaldzka 6, 60-780 Poznań, Poland

## Abstract

The absolute configuration of the title compound, C_20_H_23_NO_2_, has been confirmed as 4*S*. The benzyl residue and H atom at the asymmetric C-atom centre occupy pseudo-axial and bis­ectional positions, respectively. The oxazolidine ring adopts an envelope conformation. In the crystal structure, the mol­ecular packing is stabilized by non-classical C—H⋯O hydrogen bonds.

## Related literature

For details of the synthesis, see: Chrzanowska & Dreas (2004 ▶); Chrzanowska *et al.* (2005 ▶). For bond-length data, see: Allen *et al.* (1987 ▶). For a description of the Cambridge Structural Database, see: Allen (2002 ▶). For ring puckering parameters, see: Cremer & Pople (1975 ▶).
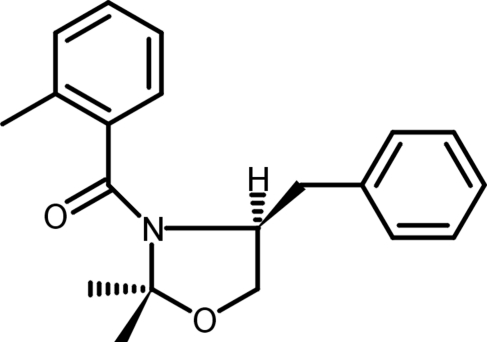


## Experimental

### 

#### Crystal data

C_20_H_23_NO_2_
*M* *_r_* = 309.39
Orthorhombic, 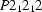

*a* = 10.9951 (2) Å
*b* = 17.2768 (3) Å
*c* = 9.1899 (2) Å
*V* = 1745.71 (6) Å^3^
*Z* = 4
Cu *K*α radiation
μ = 0.59 mm^−1^
*T* = 130 K
0.30 × 0.20 × 0.09 mm

#### Data collection

Oxford Diffraction SuperNova Single source at offset Atlas diffractometer
Absorption correction: multi-scan (*CrysAlis PRO*; Oxford Diffraction, 2009 ▶) *T*_min_ = 0.882, *T*_max_ = 1.000
9043 measured reflections
3332 independent reflections
3315 reflections with *I* > 2σ(*I*)
*R*_int_ = 0.013

#### Refinement

*R*[*F* ^2^ > 2σ(*F* ^2^)] = 0.027
*wR*(*F* ^2^) = 0.073
*S* = 1.05
3332 reflections
211 parameters
H-atom parameters constrained
Δρ_max_ = 0.14 e Å^−3^
Δρ_min_ = −0.13 e Å^−3^
Absolute structure: Flack (1983 ▶), 1301 Friedel pairs
Flack parameter: 0.11 (16)

### 

Data collection: *CrysAlis PRO* (Oxford Diffraction, 2009 ▶); cell refinement: *CrysAlis PRO*; data reduction: *CrysAlis PRO*; program(s) used to solve structure: *SHELXS97* (Sheldrick, 2008 ▶); program(s) used to refine structure: *SHELXL97* (Sheldrick, 2008 ▶); molecular graphics: *ORTEP-3 for Windows* (Farrugia, 1997 ▶); software used to prepare material for publication: *WinGX* (Farrugia, 1999 ▶) and *PLATON* (Spek, 2009 ▶).

## Supplementary Material

Crystal structure: contains datablocks I, global. DOI: 10.1107/S1600536810040699/bt5373sup1.cif

Structure factors: contains datablocks I. DOI: 10.1107/S1600536810040699/bt5373Isup2.hkl

Additional supplementary materials:  crystallographic information; 3D view; checkCIF report

## Figures and Tables

**Table 1:** Hydrogen-bond geometry (Å, °)

*D*—H⋯*A*	*D*—H	H⋯*A*	*D*⋯*A*	*D*—H⋯*A*
C4—H4⋯O9^i^	0.98	2.54	3.4487 (13)	154

Symmetry code: (i) 
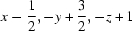
.

**Fig. 1.**
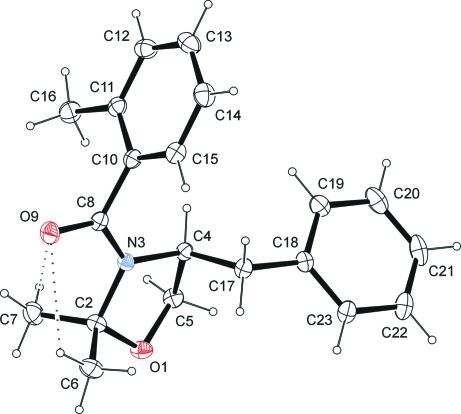
The molecular structure of (I), showing the atomic labelling scheme. Non-H atoms are drawn as 30% probability displacement ellipsoids; H atoms are shown as small spheres of arbitrary radius.

**Fig. 2.**
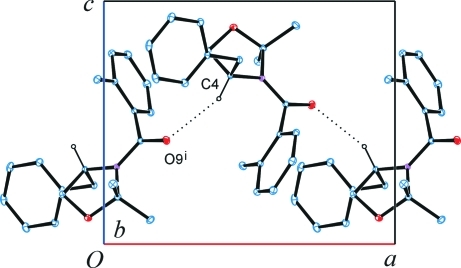
The hydrogen bonding in the crystal structure of (I). Dotted lines indicate hydrogen bonds. Symmetry code: (i) -1/2 + x, 1.5 - y, 1 - z. The H atoms not involved in hydrogen bonds have been omitted for clarity.

## supplementary crystallographic information

### Comment

(4*S*)-2,2-Dimethyl-3-*o*-toluoyl-4-benzyloxazolidine has been succesfully used as a building block and chiral auxiliary in the asymmetric synthesis of (*S*)-(-)-*O*-methylbharatamine, a protoberberine derivative. The key step of the synthesis, in which a new stereogenic centre was created, involved the addition of latherally lithiated chiral *o*-toluamide to imine. This addition proceeded stereoselectively for cyclic imine as well as acyclic imine (Chrzanowska & Dreas, 2004); Chrzanowska *et al.*, 2005). The title chiral *o*-toluamide was obtained from commercially available *o*-toluoyl chloride and (*S*)-phenylalaninol, followed by protection of functional NH and OH groups in the form of oxazolidine derivative. In order to obtain confirmation of the absolute configuration of the title compound, a single X-ray diffraction study has been undertaken.

The results obtained for the title compound confirm the absolute 4*S* configuration (Fig. 1). The benzyl residue and H atom at the stereogenic C4 centre occupy a pseudo-axial and bisectional positions, respectively, as seen in the angles of the C4—C17 and C4—H4 bond vectors to the Cremer & Pople oxazolidine ring plane normal of 16.52 (7)° and 53.11 (4)° (Cremer & Pople, 1975).

The mutual arrangement of the benzyl and oxazolidine systems is described by the torsion angles C18—C17—C4—N3 177.06 (8)° and C18—C17—C4—C5 66.22 (11)° indicating an antiperiplanar and synclinal conformation of the C18 atom in the phenyl group with respect to the N3 and C5 atom of the oxazolidine ring, respectively. Furthermore, the dihedral angle made by the mean planes of the above mentioned six- and five-membered systems amounts to 49.66 (4)°.

In the solid state, the   oxazolidine ring has an envelope conformation [puckering parameters (Cremer & Pople, 1975) Q = 0.379 (1) Å and φ = 330.13 (16)°], with atom C5 deviating from the planar system defined by the other four atoms by 0.5776 (15) Å.

The C=O group of the amide group is synperiplanar with respect to the C2—N3 bond [the torsion angle O9—C8—N3—C2: 3.59 (15)°]. We assume that this atom arrangement is stabilized by the three-centre intramolecular C6—H6B···O9···H7B—C7 hydrogen bond (Fig. 1, Table 1). The nearly planar tertiary amide group (C2/N3/C4/C8/O9, r.m.s. = 0.010) and the benzene ring (C10—C15) are not conjugated, the dihedral angle between their mean planes being 88.60 (3)°. Simultaneously, the C8—C10 bond distance of 1.5060 (14) Å is comparable with the normal length of the unconjugated (*N—*)C(=O)—C*_ar_* bond of 1.500 (5) Å (Allen *et al.*, 1987). The C8–N3 bond distance of 1.3460 (13) Å is the same as the normal C—N tertiary amide distance [1.346 (5) Å; Allen *et al.*, 1987].

The internal bond lengths and angles in 2,2-dimethyloxazolidine ring are close to those observed in other dimethyloxazolidine derivatives denoted by the following refcodes EBETUA, PIBSAV, VUMMOF, VUMNEW, WAVPUE (CSD Cambridge, version 5.31, Allen, 2002; *R*≤ 0.050).

The molecular packing in the crystal lattice is stabilized by possible C4—H4···O9^i^ non-classical intermolecular hydrogen bonds which links molecules into chains parallel to the *b* axis. (Fig. 2, Table 1).

### Experimental

(4*S*)-2,2-Dimethyl-3-*o*-toluoyl-4-benzyloxazolidine has been synthesized from *o*-toluoyl chloride and (*S*)-phenylalaninol, according to literature procedure of Chrzanowska and Dreas (2004). Crystals were obtained after crystallization from diethyl ether, mp. 361—363 K, [α]_D_ = -36.35 [*c* 1.0, CHCl_3_].

### Refinement

All H atoms were positioned geometrically and were refined in a riding-model approximation with *U*_iso_ constrained to be 1.2 (1.5 for methyl groups) times *U*_eq_ of the parent atom. The methyl H atoms were refined as rigid groups, which were allowed to rotate. The range of C—H distances was 0.93–0.98 Å. The absolute configuration of the title compound was established by refinement of the Flack (1983) parameter. The rather large s.u. of the Flack parameter is due to the small contribution of atoms with measurable anomalous dispersion effects; refinement of the inverse structure leads to a value close to 1 [*x* = 0.89 (16)], which provides additional proof of the correct assignment of the absolute configuration.

### Crystal data

**Table:**

C_20_H_23_NO_2_	*D*_x_ = 1.177 Mg m^−^^3^
*M**_r_* = 309.39	Melting point = 361–363 K
Orthorhombic, *P*2_1_2_1_2	Cu *K*α radiation, λ = 1.54184 Å
Hall symbol: P 2 2ab	Cell parameters from 8954 reflections
*a* = 10.9951 (2) Å	θ = 2.6–75.3°
*b* = 17.2768 (3) Å	µ = 0.59 mm^−^^1^
*c* = 9.1899 (2) Å	*T* = 130 K
*V* = 1745.71 (6) Å^3^	Prism, colourless
*Z* = 4	0.30 × 0.20 × 0.09 mm
*F*(000) = 664	

### Data collection

**Table:**

Oxford Diffraction SuperNova Single source at offset Atlas diffractometer	3332 independent reflections
Radiation source: SuperNova (Cu) X-ray Source	3315 reflections with *I* > 2σ(*I*)
mirror	*R*_int_ = 0.013
Detector resolution: 5.2679 pixels mm^-1^	θ_max_ = 75.5°, θ_min_ = 4.8°
ω scans	*h* = −9→13
Absorption correction: multi-scan (*CrysAlis PRO*; Oxford Diffraction, 2009)	*k* = −21→21
*T*_min_ = 0.882, *T*_max_ = 1.000	*l* = −11→11
9043 measured reflections	

### Refinement

**Table:**

Refinement on *F*^2^	Secondary atom site location: difference Fourier map
Least-squares matrix: full	Hydrogen site location: inferred from neighbouring sites
*R*[*F*^2^ > 2σ(*F*^2^)] = 0.027	H-atom parameters constrained
*wR*(*F*^2^) = 0.073	*w* = 1/[σ^2^(*F*_o_^2^) + (0.0438*P*)^2^ + 0.2282*P*] where *P* = (*F*_o_^2^ + 2*F*_c_^2^)/3
*S* = 1.05	(Δ/σ)_max_ = 0.001
3332 reflections	Δρ_max_ = 0.14 e Å^−^^3^
211 parameters	Δρ_min_ = −0.13 e Å^−^^3^
0 restraints	Absolute structure: Flack (1983), 1301 Friedel pairs
Primary atom site location: structure-invariant direct methods	Flack parameter: 0.11 (16)

### Special details

**Table:**

Experimental. Absorption correction: CrysAlis Pro (Oxford Diffraction, 2009); Empirical absorption correction using spherical harmonics, implemented in SCALE3 ABSPACK scaling algorithm.
Geometry. All e.s.d.'s (except the e.s.d. in the dihedral angle between two l.s. planes) are estimated using the full covariance matrix. The cell e.s.d.'s are taken into account individually in the estimation of e.s.d.'s in distances, angles and torsion angles; correlations between e.s.d.'s in cell parameters are only used when they are defined by crystal symmetry. An approximate (isotropic) treatment of cell e.s.d.'s is used for estimating e.s.d.'s involving l.s. planes.
Refinement. Refinement of *F*^2^ against ALL reflections. The weighted *R*-factor *wR* and goodness of fit *S* are based on *F*^2^, conventional *R*-factors *R* are based on *F*, with *F* set to zero for negative *F*^2^. The threshold expression of *F*^2^ > σ(*F*^2^) is used only for calculating *R*-factors(gt) *etc*. and is not relevant to the choice of reflections for refinement. *R*-factors based on *F*^2^ are statistically about twice as large as those based on *F*, and *R*- factors based on ALL data will be even larger.

### Fractional atomic coordinates and isotropic or equivalent isotropic displacement parameters (Å^2^)

**Table:**

	*x*	*y*	*z*	*U*_iso_*/*U*_eq_	
O1	0.44508 (7)	0.78744 (5)	0.89260 (8)	0.03235 (18)	
C2	0.54700 (10)	0.81054 (6)	0.80707 (11)	0.0279 (2)	
N3	0.54307 (8)	0.75492 (5)	0.68336 (9)	0.02318 (18)	
C4	0.43450 (9)	0.70515 (6)	0.69370 (11)	0.0233 (2)	
H4	0.3950	0.6997	0.5986	0.028*	
C5	0.35771 (10)	0.75411 (6)	0.79567 (12)	0.0289 (2)	
H5A	0.3142	0.7939	0.7426	0.035*	
H5B	0.2996	0.7224	0.8482	0.035*	
C6	0.65962 (11)	0.80040 (7)	0.90053 (13)	0.0381 (3)	
H6A	0.6516	0.8307	0.9876	0.057*	
H6B	0.7299	0.8173	0.8473	0.057*	
H6C	0.6687	0.7468	0.9259	0.057*	
C7	0.53116 (12)	0.89341 (7)	0.75407 (14)	0.0384 (3)	
H7A	0.4563	0.8977	0.7010	0.058*	
H7B	0.5979	0.9070	0.6917	0.058*	
H7C	0.5293	0.9278	0.8361	0.058*	
C8	0.62546 (9)	0.75504 (6)	0.57504 (11)	0.0249 (2)	
O9	0.71397 (7)	0.79867 (5)	0.57306 (9)	0.03526 (19)	
C10	0.60464 (9)	0.69888 (6)	0.45222 (11)	0.0259 (2)	
C11	0.53690 (10)	0.72128 (6)	0.33042 (11)	0.0280 (2)	
C12	0.52701 (11)	0.66930 (7)	0.21500 (12)	0.0351 (3)	
H12	0.4831	0.6833	0.1327	0.042*	
C13	0.58144 (11)	0.59718 (8)	0.22089 (15)	0.0399 (3)	
H13	0.5733	0.5632	0.1430	0.048*	
C14	0.64789 (12)	0.57531 (7)	0.34174 (14)	0.0385 (3)	
H14	0.6842	0.5267	0.3455	0.046*	
C15	0.65998 (10)	0.62643 (6)	0.45739 (13)	0.0318 (2)	
H15	0.7052	0.6122	0.5386	0.038*	
C16	0.47766 (11)	0.79954 (7)	0.32189 (13)	0.0365 (3)	
H16A	0.4368	0.8048	0.2301	0.055*	
H16B	0.5386	0.8391	0.3307	0.055*	
H16C	0.4197	0.8048	0.3994	0.055*	
C17	0.46564 (9)	0.62539 (6)	0.75855 (12)	0.0273 (2)	
H17A	0.5233	0.5996	0.6951	0.033*	
H17B	0.5046	0.6328	0.8522	0.033*	
C18	0.35598 (10)	0.57400 (6)	0.77822 (12)	0.0260 (2)	
C19	0.27740 (11)	0.55825 (7)	0.66370 (13)	0.0346 (3)	
H19	0.2917	0.5800	0.5727	0.042*	
C20	0.17747 (12)	0.51009 (7)	0.68425 (16)	0.0432 (3)	
H20	0.1252	0.5003	0.6069	0.052*	
C21	0.15498 (11)	0.47678 (6)	0.81733 (17)	0.0428 (3)	
H21	0.0885	0.4442	0.8298	0.051*	
C22	0.23189 (11)	0.49212 (7)	0.93230 (16)	0.0413 (3)	
H22	0.2174	0.4698	1.0227	0.050*	
C23	0.33108 (10)	0.54099 (6)	0.91312 (13)	0.0331 (2)	
H23	0.3816	0.5518	0.9917	0.040*	

### Atomic displacement parameters (Å^2^)

**Table:**

	*U*^11^	*U*^22^	*U*^33^	*U*^12^	*U*^13^	*U*^23^
O1	0.0323 (4)	0.0396 (4)	0.0251 (4)	0.0008 (3)	0.0023 (3)	−0.0044 (3)
C2	0.0275 (5)	0.0295 (5)	0.0267 (5)	−0.0001 (4)	−0.0019 (4)	−0.0035 (4)
N3	0.0208 (4)	0.0241 (4)	0.0246 (4)	−0.0013 (3)	−0.0005 (3)	0.0001 (3)
C4	0.0186 (5)	0.0263 (5)	0.0249 (4)	−0.0017 (4)	−0.0005 (4)	0.0020 (4)
C5	0.0232 (5)	0.0333 (5)	0.0301 (5)	0.0017 (4)	0.0027 (4)	0.0000 (4)
C6	0.0360 (6)	0.0437 (6)	0.0346 (6)	−0.0030 (5)	−0.0113 (5)	−0.0059 (5)
C7	0.0435 (7)	0.0281 (5)	0.0437 (6)	0.0016 (5)	−0.0014 (6)	−0.0028 (5)
C8	0.0214 (5)	0.0265 (4)	0.0267 (5)	0.0005 (4)	−0.0007 (4)	0.0044 (4)
O9	0.0286 (4)	0.0387 (4)	0.0385 (4)	−0.0101 (3)	0.0048 (3)	−0.0005 (4)
C10	0.0216 (5)	0.0292 (5)	0.0267 (5)	−0.0013 (4)	0.0054 (4)	0.0018 (4)
C11	0.0235 (5)	0.0316 (5)	0.0287 (5)	−0.0025 (4)	0.0028 (4)	0.0025 (4)
C12	0.0305 (6)	0.0446 (6)	0.0302 (5)	−0.0032 (5)	−0.0006 (5)	−0.0029 (5)
C13	0.0387 (7)	0.0406 (6)	0.0405 (7)	−0.0029 (5)	0.0039 (5)	−0.0132 (5)
C14	0.0374 (6)	0.0311 (5)	0.0471 (7)	0.0041 (5)	0.0071 (6)	−0.0036 (5)
C15	0.0295 (5)	0.0319 (5)	0.0340 (6)	0.0040 (5)	0.0036 (4)	0.0030 (4)
C16	0.0356 (6)	0.0368 (6)	0.0372 (6)	0.0035 (5)	−0.0058 (5)	0.0040 (5)
C17	0.0218 (5)	0.0286 (5)	0.0313 (5)	0.0000 (4)	−0.0004 (4)	0.0047 (4)
C18	0.0220 (5)	0.0220 (4)	0.0341 (5)	0.0027 (4)	0.0012 (4)	−0.0007 (4)
C19	0.0346 (6)	0.0323 (5)	0.0370 (6)	−0.0013 (4)	−0.0036 (5)	−0.0028 (5)
C20	0.0326 (6)	0.0342 (6)	0.0627 (8)	−0.0016 (5)	−0.0125 (6)	−0.0102 (6)
C21	0.0258 (5)	0.0251 (5)	0.0774 (9)	−0.0036 (4)	0.0050 (6)	−0.0030 (5)
C22	0.0361 (6)	0.0337 (6)	0.0542 (7)	−0.0049 (5)	0.0087 (6)	0.0100 (6)
C23	0.0298 (6)	0.0315 (5)	0.0379 (6)	−0.0022 (5)	−0.0004 (5)	0.0071 (5)

### Geometric parameters (Å, °)

**Table:**

O1—C2	1.4258 (13)	C12—C13	1.3834 (18)
O1—C5	1.4311 (13)	C12—H12	0.9300
C2—N3	1.4892 (13)	C13—C14	1.3820 (18)
C2—C6	1.5172 (15)	C13—H13	0.9300
C2—C7	1.5224 (15)	C14—C15	1.3882 (17)
N3—C8	1.3460 (13)	C14—H14	0.9300
N3—C4	1.4742 (12)	C15—H15	0.9300
C4—C5	1.5187 (14)	C16—H16A	0.9600
C4—C17	1.5398 (14)	C16—H16B	0.9600
C4—H4	0.9800	C16—H16C	0.9600
C5—H5A	0.9700	C17—C18	1.5082 (14)
C5—H5B	0.9700	C17—H17A	0.9700
C6—H6A	0.9600	C17—H17B	0.9700
C6—H6B	0.9600	C18—C19	1.3886 (16)
C6—H6C	0.9600	C18—C23	1.3918 (15)
C7—H7A	0.9600	C19—C20	1.3911 (17)
C7—H7B	0.9600	C19—H19	0.9300
C7—H7C	0.9600	C20—C21	1.374 (2)
C8—O9	1.2310 (12)	C20—H20	0.9300
C8—C10	1.5060 (14)	C21—C22	1.379 (2)
C10—C15	1.3926 (15)	C21—H21	0.9300
C10—C11	1.3990 (15)	C22—C23	1.3905 (16)
C11—C12	1.3940 (16)	C22—H22	0.9300
C11—C16	1.5029 (16)	C23—H23	0.9300
			
C2—O1—C5	107.28 (7)	C13—C12—C11	121.11 (11)
O1—C2—N3	102.56 (8)	C13—C12—H12	119.4
O1—C2—C6	107.28 (9)	C11—C12—H12	119.4
N3—C2—C6	112.42 (9)	C14—C13—C12	120.42 (11)
O1—C2—C7	110.47 (10)	C14—C13—H13	119.8
N3—C2—C7	111.06 (9)	C12—C13—H13	119.8
C6—C2—C7	112.53 (10)	C13—C14—C15	119.47 (11)
C8—N3—C4	126.41 (8)	C13—C14—H14	120.3
C8—N3—C2	122.96 (8)	C15—C14—H14	120.3
C4—N3—C2	110.53 (8)	C14—C15—C10	120.25 (11)
N3—C4—C5	99.50 (8)	C14—C15—H15	119.9
N3—C4—C17	111.52 (8)	C10—C15—H15	119.9
C5—C4—C17	112.53 (9)	C11—C16—H16A	109.5
N3—C4—H4	110.9	C11—C16—H16B	109.5
C5—C4—H4	110.9	H16A—C16—H16B	109.5
C17—C4—H4	110.9	C11—C16—H16C	109.5
O1—C5—C4	103.59 (8)	H16A—C16—H16C	109.5
O1—C5—H5A	111.0	H16B—C16—H16C	109.5
C4—C5—H5A	111.0	C18—C17—C4	113.29 (8)
O1—C5—H5B	111.0	C18—C17—H17A	108.9
C4—C5—H5B	111.0	C4—C17—H17A	108.9
H5A—C5—H5B	109.0	C18—C17—H17B	108.9
C2—C6—H6A	109.5	C4—C17—H17B	108.9
C2—C6—H6B	109.5	H17A—C17—H17B	107.7
H6A—C6—H6B	109.5	C19—C18—C23	118.19 (10)
C2—C6—H6C	109.5	C19—C18—C17	121.46 (10)
H6A—C6—H6C	109.5	C23—C18—C17	120.35 (10)
H6B—C6—H6C	109.5	C18—C19—C20	120.38 (12)
C2—C7—H7A	109.5	C18—C19—H19	119.8
C2—C7—H7B	109.5	C20—C19—H19	119.8
H7A—C7—H7B	109.5	C21—C20—C19	120.90 (12)
C2—C7—H7C	109.5	C21—C20—H20	119.6
H7A—C7—H7C	109.5	C19—C20—H20	119.6
H7B—C7—H7C	109.5	C20—C21—C22	119.41 (11)
O9—C8—N3	122.95 (10)	C20—C21—H21	120.3
O9—C8—C10	120.24 (9)	C22—C21—H21	120.3
N3—C8—C10	116.81 (8)	C21—C22—C23	120.03 (12)
C15—C10—C11	120.57 (10)	C21—C22—H22	120.0
C15—C10—C8	119.15 (9)	C23—C22—H22	120.0
C11—C10—C8	120.15 (9)	C22—C23—C18	121.07 (11)
C12—C11—C10	118.17 (10)	C22—C23—H23	119.5
C12—C11—C16	120.40 (10)	C18—C23—H23	119.5
C10—C11—C16	121.42 (10)		
			
C5—O1—C2—N3	−28.52 (10)	C15—C10—C11—C12	−0.25 (15)
C5—O1—C2—C6	−147.11 (9)	C8—C10—C11—C12	175.64 (9)
C5—O1—C2—C7	89.91 (10)	C15—C10—C11—C16	−179.28 (10)
O1—C2—N3—C8	−179.07 (9)	C8—C10—C11—C16	−3.39 (15)
C6—C2—N3—C8	−64.17 (13)	C10—C11—C12—C13	0.67 (16)
C7—C2—N3—C8	62.91 (13)	C16—C11—C12—C13	179.71 (11)
O1—C2—N3—C4	4.37 (10)	C11—C12—C13—C14	−0.47 (18)
C6—C2—N3—C4	119.27 (10)	C12—C13—C14—C15	−0.17 (19)
C7—C2—N3—C4	−113.65 (10)	C13—C14—C15—C10	0.58 (18)
C8—N3—C4—C5	−157.19 (9)	C11—C10—C15—C14	−0.37 (16)
C2—N3—C4—C5	19.22 (10)	C8—C10—C15—C14	−176.31 (10)
C8—N3—C4—C17	83.88 (12)	N3—C4—C17—C18	177.06 (8)
C2—N3—C4—C17	−99.71 (9)	C5—C4—C17—C18	66.22 (11)
C2—O1—C5—C4	41.88 (10)	C4—C17—C18—C19	53.80 (13)
N3—C4—C5—O1	−35.86 (9)	C4—C17—C18—C23	−126.17 (10)
C17—C4—C5—O1	82.31 (10)	C23—C18—C19—C20	−0.61 (16)
C4—N3—C8—O9	179.58 (9)	C17—C18—C19—C20	179.41 (10)
C2—N3—C8—O9	3.59 (15)	C18—C19—C20—C21	−0.49 (18)
C4—N3—C8—C10	0.08 (14)	C19—C20—C21—C22	0.75 (18)
C2—N3—C8—C10	−175.92 (9)	C20—C21—C22—C23	0.10 (18)
O9—C8—C10—C15	86.97 (13)	C21—C22—C23—C18	−1.22 (18)
N3—C8—C10—C15	−93.51 (11)	C19—C18—C23—C22	1.46 (16)
O9—C8—C10—C11	−88.98 (12)	C17—C18—C23—C22	−178.56 (10)
N3—C8—C10—C11	90.54 (11)		

### Hydrogen-bond geometry (Å, °)

**Table:**

*D*—H···*A*	*D*—H	H···*A*	*D*···*A*	*D*—H···*A*
C4—H4···O9^i^	0.98	2.54	3.4487 (13)	154
C6—H6B···O9	0.96	2.55	3.0683 (15)	114
C7—H7B···O9	0.96	2.51	3.0800 (15)	118

Symmetry codes: (i) *x*−1/2, −*y*+3/2, −*z*+1.
